# Functional Characterization of Novel *ATP7B* Variants for Diagnosis of Wilson Disease

**DOI:** 10.3389/fped.2018.00106

**Published:** 2018-04-30

**Authors:** Sarah Guttmann, Friedrich Bernick, Magdalena Naorniakowska, Ulf Michgehl, Sara Reinartz Groba, Piotr Socha, Andree Zibert, Hartmut H. Schmidt

**Affiliations:** ^1^Medizinische Klinik B für Gastroenterologie und Hepatologie, Universitätsklinikum Münster, Münster, Germany; ^2^Department of Gastroenterology, Hepatology, Nutritional Disorders and Pediatrics, The Children's Memorial Health Institute, Warsaw, Poland; ^3^Internal Medicine D, Molecular Nephrology, University Hospital of Münster, Münster, Germany

**Keywords:** delay of diagnosis, copper, neuropsychiatric, WD scoring, rare disease, cell model

## Abstract

**Background:** Diagnosis of rare Wilson disease (WD) in pediatric patients is difficult, in particular when hepatic manifestation is absent. Genetic analysis of *ATP7B* represents the single major determinant of the diagnostic scoring system in WD children having mild symptoms.

**Objectives:** To assess the impact of molecularly expressed ATP7B gene products in order to assist diagnosis of Wilson disease in pediatric patients having a novel mutation and subtle neuropsychiatric disease.

**Methods:** The medical history, clinical presentation, biochemical parameters, and the genetic analysis of *ATP7B* were determined. Due to ambiguous clinical and biochemical findings and identification of a novel compound *ATP7B* mutation with unknown disease-causing status, a molecular analysis of the ATP7B gene products in a previously well characterized cell model was performed.

**Results:** The *ATP7B* variants were transgenically expressed and the respective gene function molecularly characterized. Despite normal mRNA expression, low ATP7B protein expression of the mutants p.L168P and p.S1423N was observed (34.3 ± 8% and 66.0 ± 8%, respectively). Copper exposure did not result in decreased viability of transgenic cells as compared to wild type. Intracellular copper accumulation was reduced (≤47.9 ± 8%) and intracellular protein trafficking was impaired.

**Conclusion:** Our report suggests that functional characterization of novel ATP7B mutants can assist diagnosis; however mild functional impairments of ATP7B variants may hamper the value of such approaches.

## Introduction

Wilson disease (WD; MIM#277900) is a rare, autosomal recessive, monogenetic disorder with a frequency of approximately 1:30,000 [1]. Diagnosis of WD represents a challenge to doctors, including pediatricians [2–4]. The disease manifests at various times throughout life with children representing a major portion of patients. Commonly, disease is diagnosed between 5 and 18 years, however some children below 5 years and elderly (> 60 years) can show first symptoms [5, 6]. WD results from mutations in the *ATP7B* gene. The gene encodes a large membrane-spanning P-type ATPase [7, 8]. *ATP7B* is predominately expressed in hepatocytes where it has two main functions: it is responsible for transfer of copper to apoceruloplasmin, which is secreted into the blood for copper supply to other organs, and—in case of excess copper—excretion from the body into the bile [9]. Clinical presentation with pure neurological symptoms in childhood, e.g., tremor or movement disorder, is rare, and most children show hepatic disease due to copper overload of the liver [10]. When left untreated, a severe, life-threatening disease evolves, including liver cirrhosis and/or severe neurodisability. Of note, effective, low-cost therapy has been established with copper chelators, mostly D-penicillamine (DPA) and trientine, or zinc [11]. However, therapy has to be taken lifelong and may result in side effects in a portion of the patients. An early start of the treatment in childhood is suggested to be of clinical benefit.

There is no single biochemical test for diagnosis of WD. A combination of individual assessments is indicative for diagnosis. Respective scores for diagnosis that includes various parameters of liver, including elevated levels of transaminases, and neurological disease have been developed [3, 12, 13]. One hallmark of the disease is the presence of a Kayser-Fleischer ring (KF) which is however absent in a significant portion of patients [14, 15]. Low serum ceruloplasmin and high liver copper are also highly suggestive but could be missed in patients with predominant neurological or psychiatric symptoms [16]. Significant delay of WD diagnosis is therefore not uncommon.

The only single assay to confirm WD is by genetic testing of *ATP7B*. Detection rates of up to 98% have been reported [17, 18]. More than 650 disease-causing mutations are currently known and novel SNPs are continuously reported. For novel mutations the prediction of penetrance is difficult. Most mutants of ATP7B are missense mutations which are proposed to reduce the function of the protein. To assess *ATP7B* function, primary specimens derived by biopsy from the patient are limited and also ethically restricted. Consequently, several cellular models reflecting the activity of *ATP7B* were established to predict the disease-causing impact of a given mutation. Such systems include yeast and several mammalian cell lines [19–22]. In this work we describe a case report of a child with a novel compound *ATP7B* mutation and relatively mild symptoms, where we have used a previously well characterized cell model to classify the functions of *ATP7B* for improved diagnosis of WD [23].

## Materials and methods

### Clinical presentation

Medical history, family medical history, clinical presentation, and biochemical parameters were recorded in the Department of Gastroenterology, Hepatology, Nutrition Disorders and Pediatrics of the Children's Memorial Health Institute Warsaw, Poland. Alanine transaminase (ALT), asparganine transaminase (AST), bilirubin, serum ceruloplasmin, rhodanine staining of hepatocytes, Kayser-Fleischer ring (KF), and 24 h urinary copper excretion were determined as reported [24]. After obtaining written informed consent, genomic DNA was extracted from peripheral blood samples and the complete open reading frame and adjacent intron boundaries of *ATP7B* were sequenced at the Medizinische Klinik B für Gastroenterologie und Hepatologie, Universitätsklinikum Münster, Germany. WD was diagnosed based on the Ferenci scoring system [25].

### Cell culture

HepG2 (human hepatocellular carcinoma) cells were purchased from American Type Culture Collection (ATCC) and ATP7B KO cells were derived as described [26]. RPMI medium (Lonza) containing 10% fetal bovine serum (FBS) was supplemented with 100 U/mL penicillin/streptomycin (PAA). Cells were maintained in 5% CO_2_ at 37°C in a humidified chamber.

### Site-directed mutagenesis and generation of stable ATP7B mutant cell lines

Wild type *ATP7B* cDNA was cloned into plasmid pGCsamEN.ATP7B and site-directed mutagenesis was performed using QuikChange II XL Site-Directed Mutagenesis Kit (Agilent Technologies) [23]. The primer sequences were: p.L168P (5′-3′): GGCAAGGTCCGGAAACCGCAAGGAGTAGTGAG /CTCACTACTCCTTGCGGTTTCCGGACCTTGCC and p.S1423N (5′-3′): CCATGGGACCAGGTCAACTATGTCAGCCAGGT / ACCTGGCTGACATAGTTGACCTGGTCCCATGG. Retroviral vector transduced HepG2 KO cells were selected in media containing 6 μg/ml blasticidin (Invitrogen).

### Western blot

A polyclonal anti-rabbit ATP7B antibody (kind gift of Dr. I. Sandoval, Madrid, Spain) was used for Western blot. HSC70 (Santa Cruz Biotechnology, #sc-1059) staining was used as a protein loading control. Relative expression was normalized to HepG2 KO cells expressing wild type *ATP7B* [23].

### MTT assay

For cell viability assay triplicates of 10^4^ cells per 96 well were seeded and cultivated overnight in 100 μl RPMI media lacking phenol red (Lonza). Cells were exposed to copper (CuCl_2_; Sigma Aldrich) for 48 h. Viability was determined by MTT assay [26]. The percentage of viable cells was calculated and compared to untreated (100%).

### Copper accumulation

For copper accumulation assay 10^5^ cells were seeded per 12 well, cultivated overnight and treated with 0.01 mM CuCl_2_ for 4 h. Cells were washed twice, trypsinized and lyzed in 65% nitric acid (Merck). Bradford Assay (BioRad) was used to determine total protein concentration. Analysis of copper accumulation was performed via inductively coupled plasma mass spectrometry (Thermo Fisher Scientific iCAP Qc).

### Real-time PCR analysis

RT-qPCR analysis was performed using SYBR Green PCR Core Plus (Eurogentec). Ct values were normalized to the expression of the house-keeping *GAPDH* gene (ΔΔct method). PCR analysis was conducted on the ABI Prism 7900 HT Sequence Detection System (PE Applied Biosystems). Following primer sequences were used: *ATP7B* (5′-3′): TCCTCTGTGTCTGTGGTGCTC / ATGCGCCTGTGCCTCATAC and *GAPDH* (5′-3′): CCCACTCCTCCACCTTTGAC / CCACCACCCTGTTCCTGTAG.

### Confocal staining

For confocal microscopy, cells were maintained in RPMI basal cell culture media or treated by addition of 100 μM copper for 3 h. Primary antibody staining was performed using anti-ATP7B (kind gift of Dr. I. Sandoval, Madrid, Spain) and anti-lamp2 (Santa Cruz Biotechnology, #sc-18822). Three independent experiments were performed. Microscopic images were recorded with an Observer Z1 microscope with Apotome, Axiocam MRm (Zeiss) [27].

### Statistical analysis

Statistical analysis was performed by Kruskal-Wallis 1-way ANOVA and Student's *t*-test using SPSS 22.0 software. Data are given as mean ± standard error (SE).

## Results

### Patient history

**Figure 1:**
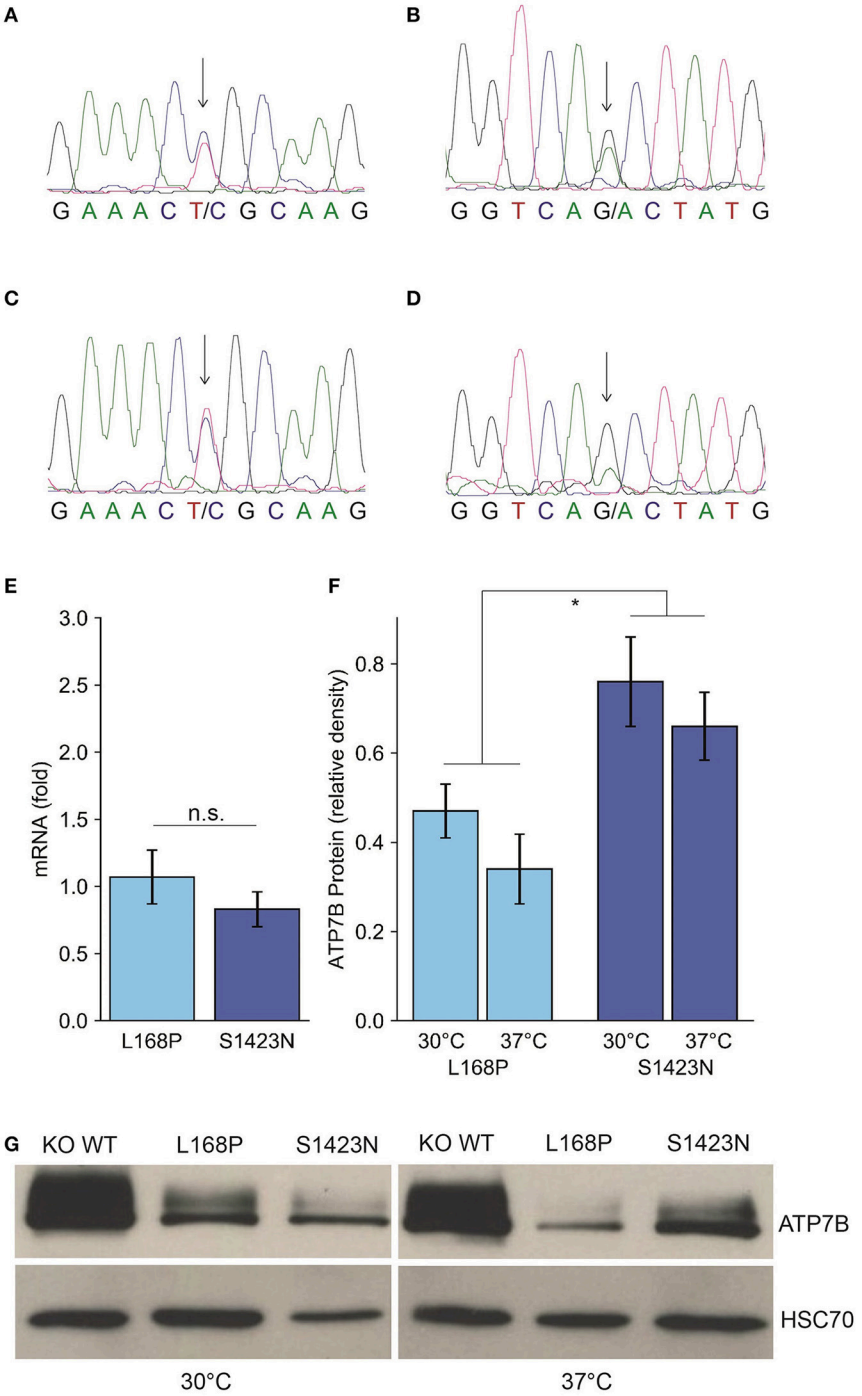
Identification and expression of ATP7B mutants p.L168P and p.S1423N. **(A**,**B)** Sequence analysis of the patient shows a compound heterozygote p.L168P **(A)** and p.S1423N **(B)** mutation. **(C**,**D)** The respective sequences derived from the father **(C)** and mother **(D)** are also depicted. **(E)** ATP7B mRNA expression of both variants was determined by RT-qPCR analysis. Mean/SE are given (*n* = 3). **(F)** Densitometry determination of ATP7B protein expression relative to wild type is shown. Mean/SE are given (*n* = 3). *P < 0.05. **(G)** One typical Western blot is shown.

The clinical characterization of the patient is illustrated in Table 1. At the age of thirteen, the boy was hospitalized following episodes of paranoia, uncommon tics, Tourette syndrome and three attempted suicides. MRI was performed, showing typical images according to age. Serum transaminases were within normal thresholds. Ceruloplasmin was low (0.13 g/L) at one occasion, while a second determination showed normal values (0.22 g/L). Liver biopsy was performed indicating micro- and macrovesicular steatosis with no signs of necrosis, fibrosis or cholestasis. Liver copper was in the normal range. Urine copper concentration was highly elevated (>5 fold) after cuprenil challenge [28]. A previously reported, disease-causing heterozygous p.L168P (c.503T > C) mutation was detected in *ATP7B* [29]. A second, unknown heterozygous *ATP7B* variant p.S1423N (c.4268G > A) was observed. The parents were shown to be asymptomatic heterozygotic carriers (Figures 1A–D). Genetic analysis of the patient also revealed a p.TA7/7 mutation in *UGT1A1* indicating Gilbert syndrome. DPA and zinc acetate treatment lead to no further reports of neurological and hepatic disease. According to the diagnosing of WD using the Ferenci scoring system an overall score of 4 was determined [25].

**Table 1:** Patient characteristics.

	**ALT AST (U/L)**	**CP (g/L)**	**Liver Cu (μg/g)**	**Liver Rhodanine**	**Urine Cu Normal/ DPA (μg/24 h)**	**Neuro-psychiatric**	**ATP7B**	**KF**	**Therapy**
2011	< 40 < 35	–	–	–	ND	Tourette^1^ Paranoia^1^ Suicide^1^	–	–	–
2013	< 40 < 35	0.13^1^ 0.22	20	absent	1,50^1^ 4,25^1^	–	–	absent	DPA
2016	< 40 < 35	–	–	–	–	–	L168P^1^ S1423N	–	Zinc
WD score^2^	–	1	−1	0	2	1	1	0	(total 4)

1 *indicating WD*.

2 *scoring according to Ferenci et al. [25]; –, not determined/applicable; DPA- d-penicillamine*.

### Expression profile of novel ATP7B variants

Since functional characterization of ATP7B mutants is not possible using patient materials and biopsy represents a risk especially for children, a previously established cellular model was used to functionally characterize the mutations of the patient [23]. While mRNA expression of the *ATP7B* variants p.L168P and p.S1423N was in the same range as observed for wild type (Figure 1E), protein expression/stability was significantly affected in both mutants of the mutants p.L168P and p.S1423N (34.3 ± 8% and 66.0 ± 8%, respectively) (Figures 1F,G). Incubation at low temperature (30°C) could marginally increase protein stability for mutants p.L168P and p.S1423N (factor of ≈1.4 and ≈1.2, respectively).

### Characterization of ATP7B mediated copper transport

**Figure 2:**
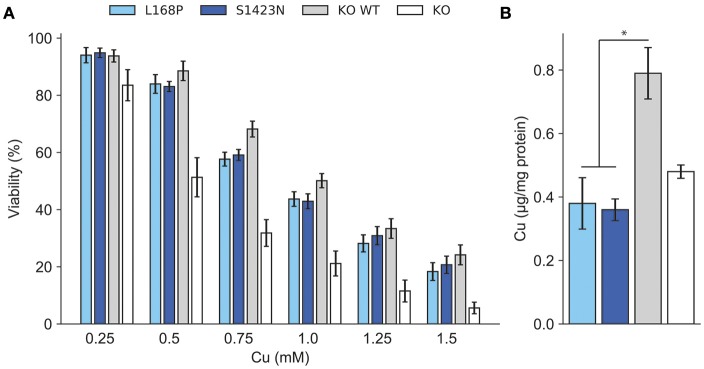
**(A)** Cell viability was determined after exposure to copper. Viability of cells relative to untreated (100%) is shown. Mean/SE are given (*n* = 3). *P < 0.05. **(B)** Analysis of intracellular Cu accumulation. Mean/SE are given (*n* = 3). *P < 0.05.

**Figure 3:**
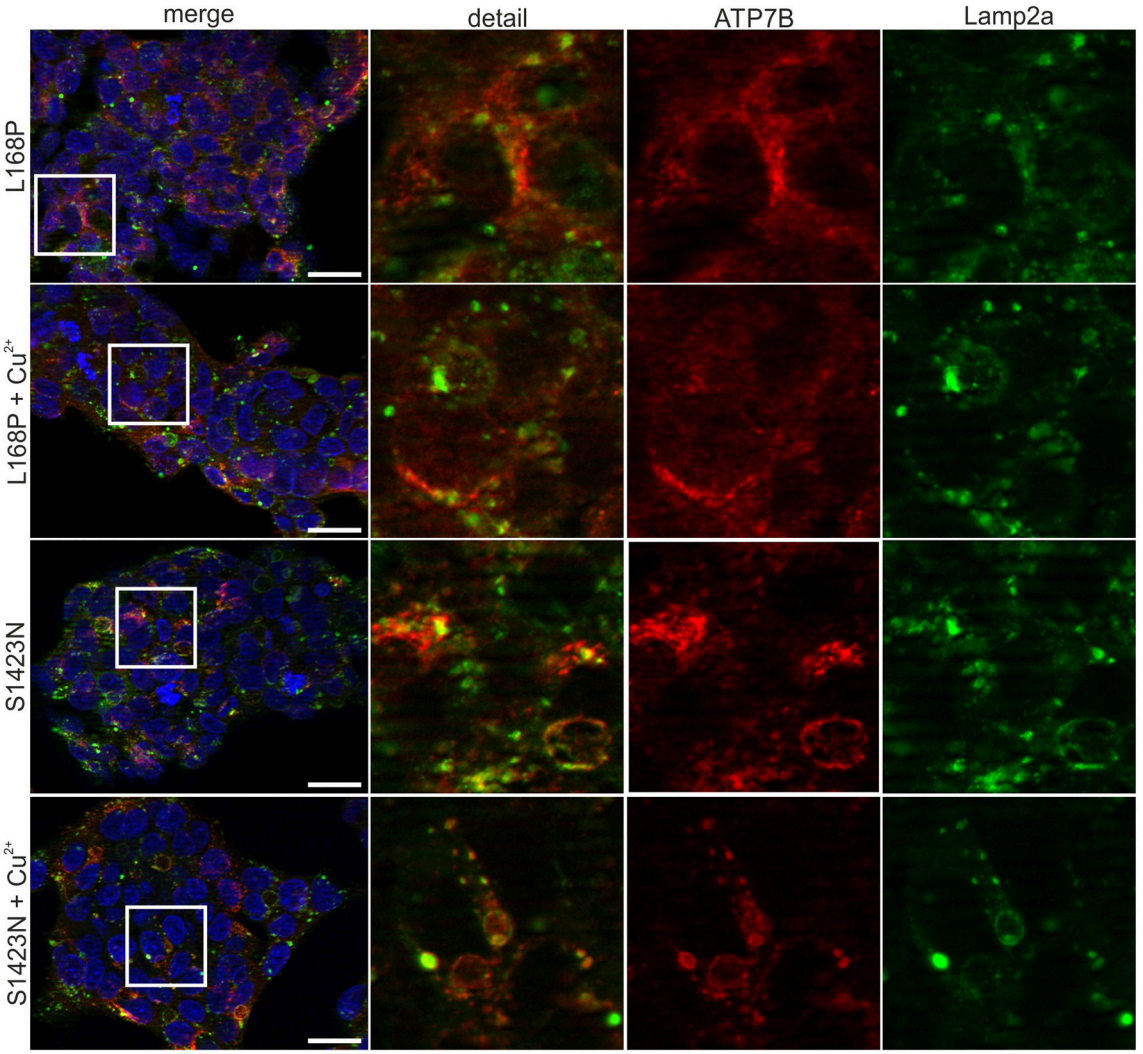
Confocal microscopy before and after addition of copper. For mutant p.L168P, a dispersed cytoplasmically staining of ATP7B was observed at low and high copper. For mutant p.S1423N, a co-localization with late endosome-lysosome marker lamp2 was observed at low copper concentrations. Elevated copper did not show typical trafficking of this mutant. One representative experiment out of three is shown. Scale bar, 20 μm.

ATP7B activity of both mutants as judged by viability of cells was similar to wild type at most copper concentrations (Figure 2A). IC50 values calculated for p.L168P and p.S1423N were similar (0.87 ± 0.06 mM and 0.89 ± 0.05 mM, respectively) and close to wild type ATP7B. The accumulated cellular copper concentration was significantly reduced (≤47.9 ± 8%) in both ATP7B mutants as compared to wild type (Figure 2B). Values of both mutants were in the same range as observed in *ATP7B* knockout cells. Confocal microscopy was used to analyze ATP7B trafficking relative to wildtype cells (Supplementary Figure S1). For mutant p.L168P, ATP7B staining was dispersed cytoplasmically at both copper concentrations (Figure 3). A broad co-localization with anti-lamp2, a late endosome-lysosome marker, as observed before for wild type was not detected [23]. For p.S1423N, a co-localization of ATP7B and lamp2 staining was found at low copper concentrations. The ATP7B staining pattern obtained with this mutant moderately responded to elevated copper.

## Discussion

We here report on the diagnosis of a boy who has a novel compound heterozygote *ATP7B* mutation, no family history of WD, and subtle neuropsychiatric symptoms without hepatic disease. In this and other cases, WD diagnosis can be delayed due to highly variable symptoms and low awareness of the disease. Symptoms are rarely observed before the age of 5 years [5, 30, 31]. In the first decade, the majority of children present with hepatic symptoms. Liver disease is often accidentally revealed by routine analysis of serum showing elevated levels of hepatic transaminases which represent important first-line indicators of WD. However, the detection rate of elevated transaminases in children having WD is variable [32, 33]. While the Ferenci scoring system for diagnosis of WD includes parameters, e.g., Kayser-Fleischer rings, which are rarely observed in young children, some of the thresholds used for scoring have been adapted to children [28]. The only single parameter confirming WD is by genetic analysis of *ATP7B*. Genetic testing was proposed for children where liver disease of unknown origin is observed [3, 5]. Irrespective of the presence of liver disease, a family history of WD is highly suggestive for genetic testing. Extrahepatic presentation of WD as observed in the case described here is uncommon at young age. In children presenting with dystonia, tremor, dysarthria and/or impaired school performance, WD should be however carefully taken into account.

The reported case is unusual with respect to a pure psychiatric disease manifestation observed in childhood [34]. All clinical, biochemical, and histological findings considered, the diagnosis of WD for the patient is tentative, since definite hallmarks of the disease, like Kayser-Fleischer ring or elevated liver copper, are missing and overall relatively mild symptoms are observed. One determination of ceruloplasmin was indicative for WD, while a second assay performed in the same year was above thresholds. Of note, around 20% of diagnosed children (and adults) have CP levels above the threshold of 20 mg/dL limiting the value of this diagnostic parameter [35–39]. Elevated urine copper after penicillamine treatment corroborated WD diagnosis. It should be mentioned however that this assay is constrained in children, since collection of urine may be difficult. Tic syndromes are rather uncommon in WD patients but have been occasionally associated with the disease [40]. In addition, the patient displayed a mutation in *UGT1A1* indicating Gilbert syndrome and total and indirect bilirubin was elevated. However, jaundice was not reported. Although we cannot fully rule out a multiple disorder of the patient, the general clinical picture seems to be in agreement with WD as the principal underlying disease. Our finding of a borderline positive WD diagnosis score could be significantly modulated by the young age of the patient which precludes a full manifestation of the disease [6, 10].

The genetic analysis of *ATP7B* in the patient revealed one previously reported disease-causing mutation p.L168P [29], while the second mutation p.S1423N was of unknown status. Variant p.S12423N was not observed in healthy individuals and could not be found in public data banks suggesting that this is not a polymorphism (data not shown). The mutation p.L168P has been reported in a 33 year old female in compound with mutation p.H1069Q, the most frequent mutation found worldwide which is associated with late-onset neurological disease [6]. The woman was diagnosed following the presentation of seizures and episodes of unconsciousness shortly after a cesarean section was performed. Neurological disease was not reported and zinc sulfate treatment resulted in functional improvement suggesting that mutation p.L168P may be associated with a relative mild course of disease. However, apart from this report, a coherent phenotype of p.L168P observed in homozygous WD patients has not been reported. While mutation p.L168P, located in the second copper binding domain of ATPase 7B, is predicted to alter the function of the protein, such analyses gave unambiguous results for p.S1423N (data not shown). The latter mutation is located in the cytosolic portion of ATPase 7B close to the C-terminal end, possibly important for protein trafficking [9].

To molecularly assess the function of the *ATP7B* gene products, we have employed a hepatic cell model, since hepatocytes represent the best studied cells where various biological functions of the copper transporter have been characterized [20, 26]. Given the almost pure neuropsychiatric symptoms of the patient, it would be interesting to re-address our findings in neuronal cell lines, which are however less established for assessment of WD to date. First, ATP7B-specific protein expression of mutants p.L168P and p.S1423N was found to be greatly reduced. The low protein expression could only be marginally increased by low temperature suggesting that in contrast to other mutations of *ATP7B*, a decreased translation might be operative [41]. Second, analysis of the cell viability in the presence of toxic copper indicates that mutants p.L168P and p.S1423N are mildly impaired with only marginal functional losses as compared to wild type. Of note, the viability assay employed here can detect various degrees of ATP7B activity, as exemplified by the moderate or deleterious loss-of-function mutations p.H1069Q/p.L795F and p.C271*, respectively [23]. Third, the assessment of intracellular copper accumulation revealed that both mutations have almost completely lost the ability to store copper and showed similar values as observed for knockout cells, whereas overexpression of ATP7B resulted in an increase of copper previously also observed in Chinese Hamster Ovary (CHO) cells [19]. This latter finding suggests that the copper accumulation assay and the viability assay may rely on distinct functions of ATPase 7B. As suggested earlier, copper is translocated to intracellular vesicles via ATP7B leading to increased copper concentrations [19]. Fourth, protein trafficking, a functional hallmark of the ATP7B protein, was significantly disturbed in both mutants. Mutant p.S1423N did not respond to high copper suggesting that the amino acid change at position 1423 might affect the nearby DKWSLL traffic signal that was found to be important for regulation of the transport between the trans-Golgi network (TGN) and the plasma membrane [42]. Trafficking of mutant p.L168P showed a copper dependent response, however lost a specific trans-Golgi localization. Our analysis of protein trafficking is however restricted, since quantification of protein localization relative to marker protein was not performed. Such quantitative analyses awaits further standardization, while different markers and experimental settings have been used [43–45]. Our molecular characterizations therefore indicate that both mutants exhibit distinct, non-overlapping impairments of ATP7B protein function which show different degrees of impact depending on the functional assay used for the determination.

Taken together, molecular characterization of novel ATP7B variants may help to functionally categorize the mutation and to assist in early WD diagnosis. The functional characterization of *ATP7B* gene products is straightforward and can be achieved within several weeks in order to initiate efficient therapy. However, when the impairment of *ATP7B* function is relative mild, as in the case reported here, such analysis can be ambiguous. In addition, our molecular analyses has to be subjected to further standardization by a large collection of mutated proteins which is far from being achieved in our and other studies [19–21, 23, 45]. The case reported here thus demonstrates current limitations of genetic analysis even when combined with advanced functional characterizations of *ATP7B* gene products. Given the evolving methodologies of high-throughput mutagenesis, improved cellular models to predict loss-of-function seem to be on the horizon, especially for monogenetic, severe disorders of childhood where efficient treatment is available.

## Ethic statement

This study was conducted according to the guidelines laid down in the Declaration of Helsinki and all procedures involving human subjects/patients were approved by the Ethics Committee of the Children's Memorial Health Institute (Children's Memorial Health Institute, Warsaw, Poland; approval granted in 2003). All patients and/or parents gave written informed consent.

## Author contributions

Study concept and design: SG, PS, HS, and AZ. Experiment and procedures: SG, FB , MN, UM, SRG, and PS. Results interpretation and drafting of the manuscript: SG, FB, UM, SRG, and AZ. Critical revision of the manuscript for important intellectual content: SG, MN, PS, HS, and AZ.

### Conflict of interest statement

The authors declare that the research was conducted in the absence of any commercial or financial relationships that could be construed as a potential conflict of interest.

## Abbreviations

WD: Wilson disease
DPA: D-Penicillamine
KF: Kayser-Fleischer ring
Cu: copper.
