# Supplementary material for: Functional Characterization of Novel ATP7B Variants for Diagnosis of Wilson Disease
Source: Front Pediatr. 2018 Apr 30;6:106. doi: 10.3389/fped.2018.00106 (PMC5937294; doi:10.3389/fped.2018.00106)
Supplement: Supplementary file 1 [file DataSheet1.DOCX]

Supplementary Material

# **Functional characterization of novel *ATP7B* variants for diagnosis of Wilson disease**

Sarah Guttmann^1^, Friedrich Bernick^1^, Magdalena Naorniakowska^2^, Ulf Michgehl^3^, Piotr Socha^3^, Andree Zibert^1^, Hartmut H. Schmidt^1*^

^1^Medizinische Klinik B für Gastroenterologie und Hepatologie, Universitätsklinikum Münster, Münster, Germany

^2^Department of Gastroenterology, Hepatology, Nutritional Disorders and Pediatrics, The Children’s Memorial Health Institute, Warsaw, Poland

^3^Internal Medicine D, Molecular Nephrology, University Hospital of Münster, Germany

***Corresponding author:** Hartmut H.-J. Schmidt

Medizinische Klinik B für Gastroenterologie und Hepatologie, Universitätsklinikum Münster

Albert-Schweitzer-Campus 1, Gebäude A14

48149 Münster

phone: +49 251 / 83 - 5 79 35, Fax: +49 251/ 83 - 5 77 71

mail: [hepar@ukmuenster.de](mailto:hepar@ukmuenster.de)

**Keywords:** delay of diagnosis, copper, neuropsychiatric, WD scoring, rare disease, cell model

**
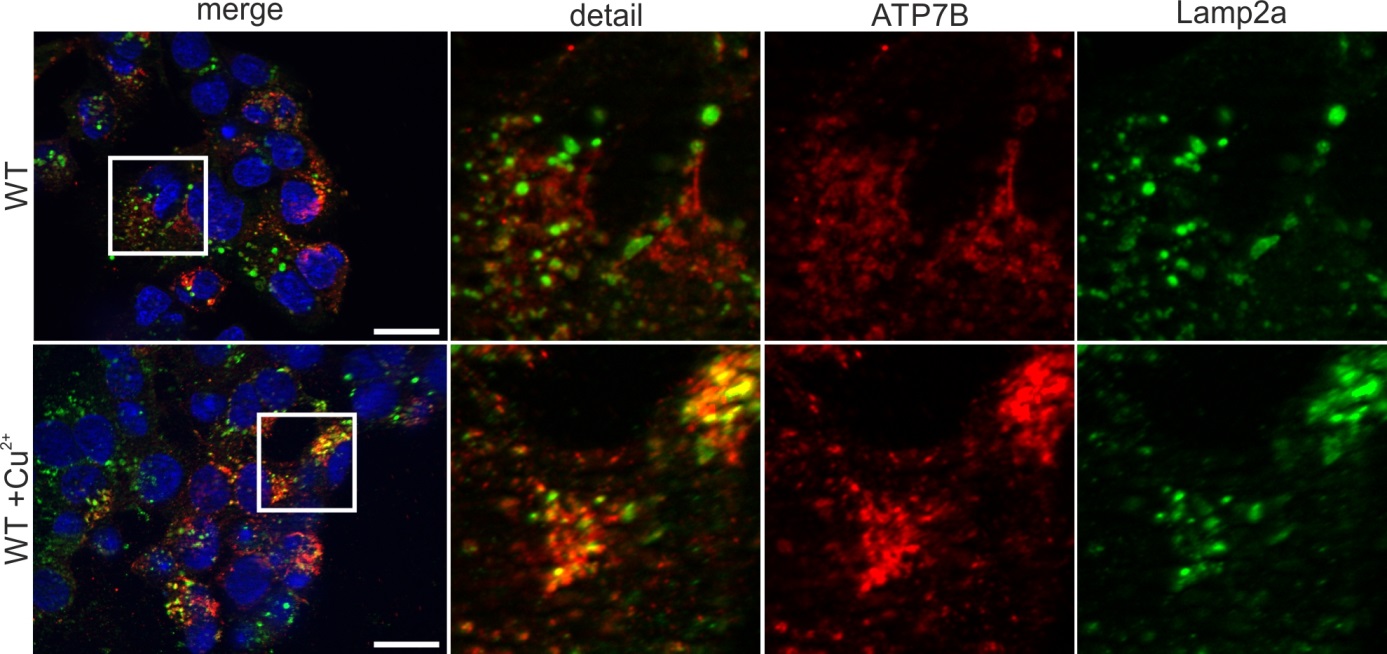
**

**Supplementary Figure S1.** Confocal microscopy of wildtype cells (WT) before and after addition of copper. Elevated copper shows co-localization with late endosome-lysosome marker lamp2. One representative experiment out of three is shown. Scale bar, 20 µm.
